# High Prevalence of and Risk Factors for Latent Tuberculosis Infection among Prisoners, Tianjin, China

**DOI:** 10.3201/eid2603.190891

**Published:** 2020-03

**Authors:** Guoqin Zhang, Yuhua Zhang, Da Zhong, Sukai Meng, Liqun An, Wenliang Wei, Zhi Zhang, Yanyong Fu, Xiexiu Wang

**Affiliations:** Tianjin Center for Tuberculosis Control, Tianjin, China (G. Zhang, Y. Zhang, D. Zhong, S. Meng, W. Wei, Z. Zhang, X. Wang);; Tianjin Centers for Disease Control and Prevention Tianjin (L. An);; Tianjin Third Central Hospital, Tianjin (Y. Fu)

**Keywords:** tuberculosis and other mycobacteria, tuberculosis, TB, latent tuberculosis, latent tuberculosis infection, respiratory infections, prevalence, risk factors, interferon-γ release assay, prisoners, prisons, Tianjin, China

## Abstract

The high incidence of tuberculosis (TB) among prisoners calls for interventions to identify latent tuberculosis infection (LTBI) before disease onset. To identify LTBI prevalence among prisoners and factors associated with it, we conducted a cross-sectional study in Tianjin. We randomly sampled 959 HIV-negative adult prisoners by ward clusters in 5 prisons and determined LTBI by seropositivity using an interferon-γ release assay. The overall rate of LTBI was 52.0% (499/959) in the 5 facilities and ranged from 41.9% (72/172) to 60.9% (106/174). Age (adjusted odds ratio [aOR] 1.7, 95% CI 1.4–2.0 per 10 years), duration of imprisonment (aOR 1.2, 95 CI% 1.1–1.2 per year), previous incarceration (aOR 2.0, 95% CI 1.5–2.7), and facility-specific TB incidence (aOR 1.9, 95% CI 1.3–2.8) were risk factors for LTBI. These findings indicate possible TB transmission within prisons and suggest the necessity for early TB case detection, as well as prophylaxis.

Tuberculosis (TB) remains a global threat to human health. In 2018, a total of 10 million cases of TB and 1.5 million deaths from TB were reported by the World Health Organization (WHO) (*1*). Globally, TB notification rates for prisoners are 11–81 times higher than those for local general populations (*2*). An estimated 10.4 million persons are held in penal institutions throughout the world, and each year the number of persons passing through prison gates could be 4–6 times higher because of high turnover of inmates (*3*). TB in prisons might spread to the civilian population through staff, visitors, and released prisoners because of inadequate treatment for TB and thus could affect TB control in the general population (*4*,*5*).

China has the second highest burden of TB in the world (0.9 million incident TB cases during 2018) (*1*). There were ≈1.7 million prisoners in China during 2016 (*3*). However, similar to the situation in other countries, TB control in prisons remained largely neglected (*6*,*7*).

Tianjin, located in northern China, has ≈16 million permanent residents and is one of the pioneering regions for TB control in China. This city had systematically implemented active TB case-finding and directly observed treatment using short-course chemotherapy in all prisons according to the guidelines (*8*,*9*).

However, despite major achievements, TB incidence among prisoners is still much higher than in the general population in Tianjin (*10*). It is essential to conduct interventions to identify latent TB infection (LTBI) through screening and prophylaxis in prisons (*11*). To achieve this goal, we conducted a study to evaluate the prevalence of and risk factors for LTBI by using an interferon-γ release assay (IGRA) in 8 prisons in Tianjin, China.

## Methods

We conducted a baseline cross-sectional study in Tianjin during 2016, followed by providing prophylaxis to prisoners with LTBI and consecutive surveillance of TB incidence. We determined the necessary sample size as >880 persons by using Epi Info (https://www.cdc.gov/epiinfo) to satisfy a cross-sectional design for estimation of LTBI (>480 persons) and a community randomized control trial design (>880 persons). The 8 prisons contained 14,401 prisoners (>18 years of age) and were composed of separate wards, each containing ≈200 persons.

Sampling was performed by using a 2-stage process and ward clustering; persons in selected wards participated in a screening procedure for the study. In the first stage of sampling, 5 prisons incarcerating 9,037 men were randomly selected in stratifications representing different levels of reported pulmonary TB incidence in the past 5 years (median 892.7 cases/100,000 persons). In the second stage of sampling, with the proviso that sample size be allocated equally among 5 prisons, 1–3 wards from 1 prison and 8 wards incarcerating persons without HIV infection were randomly selected. Screening for HIV/AIDS routinely occurred at the beginning of imprisonment for each person; HIV-positive prisoners were incarcerated in separate wards and excluded from this study.

We interviewed all participants to obtain a history of TB and treatment. We performed a chest radiograph for all participants at the beginning of the study, and further tested persons with abnormal clinical manifestation by using bacteriological tests according to WHO guidelines (*12*). The inclusion criterion was persons in selected wards with >2 years remaining in their prison terms, which enabled follow-up of pulmonary TB incidence. Exclusion criteria were HIV infection, medical history of TB (either pulmonary or extrapulmonary), anti-TB treatment for >1 month previously or currently, and refusal to participate. A total of 172–224 eligible participants from each prison (959 persons from 5 prisons) were included in the study (Figure).

**Figure:**
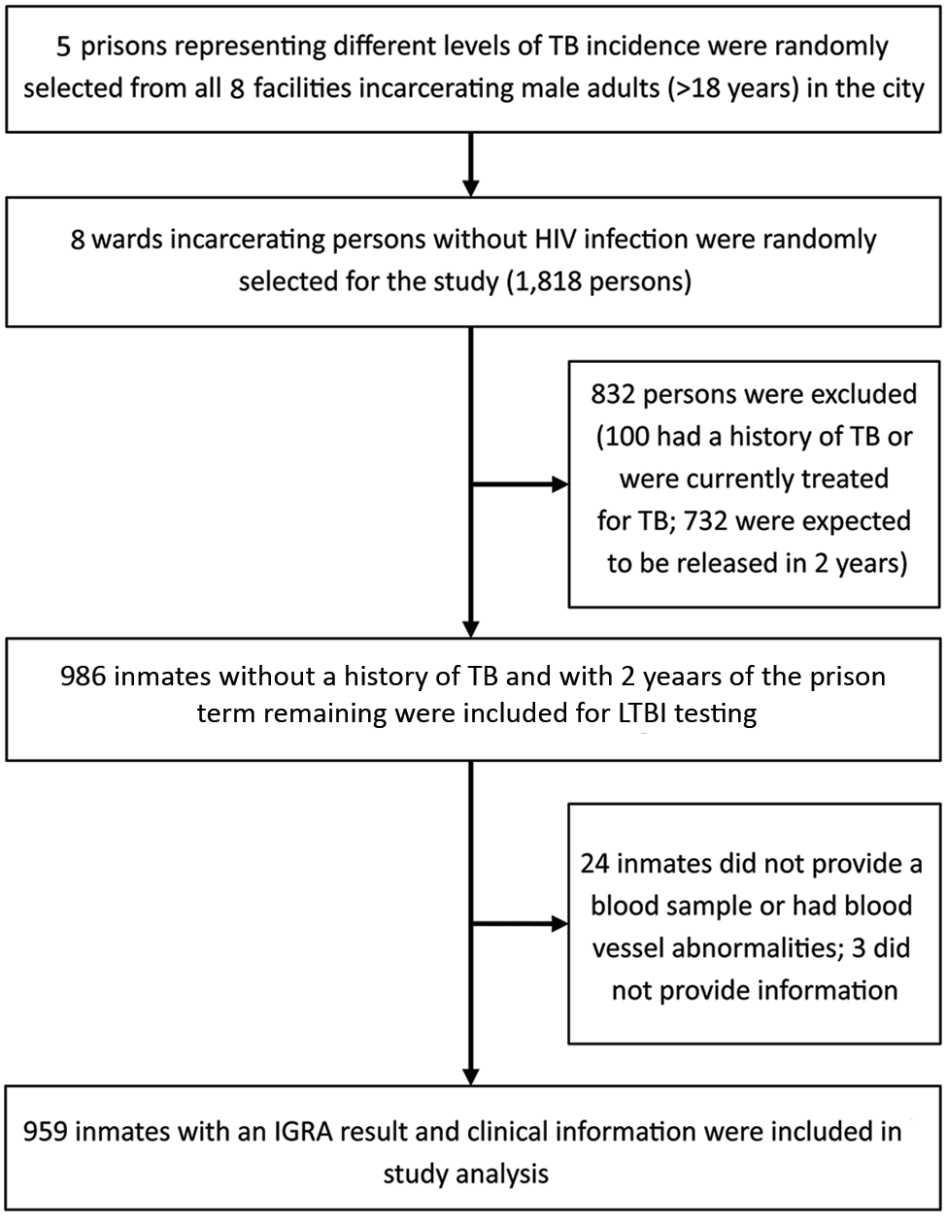
Recruitment of participants in study of high prevalence of and risk factors for latent tuberculosis among prisoners, Tianjin, China. IGRA, interferon-γ release assay; LTBI, latent tuberculosis infection; TB, tuberculosis.

We collected demographic information by using a questionnaire under the supervision of prison guards. This information included age, ethnicity, residence, employment before imprisonment, height, weight, contact history of TB, concurrent conditions other than TB (e.g., chronic diseases, such as diabetes, silicosis, hypertension, kidney disease), history of previous incarceration, and initiation of the current prison term. We calculated body mass index (BMI) as weight in kilograms divided by the square of height in meters (*13*). Physicians checked for *Mycobacterium bovis* BCG vaccine scars at the time of blood sampling. LTBI was determined by using a seropositive result in an IGRA by using the QuantiFERON-TB Gold Kit (QIAGEN, https://www.qiagen.com).

We collected questionnaires and blood samples after written consent was obtained from the participants; we then deidentified the information. Participants who were seropositive were able to receive free preventive therapy (rifampin and isoniazid) after medical evaluation once they provided consent (*11*). The study protocol was reviewed and approved by the Ethical Committee of Tianjin Centers for Disease Control and Prevention.

We compared frequencies of characteristics of participants between seropositive and seronegative persons by using conventional 2-way contingency tables and the χ^2^ test to evaluate statistical significance. We calculated odds ratios (ORs) and 95% CIs for LTBI seropositivity. We used multivariate logistic regression to analyze factors associated with seropositivity and for calculation of adjusted ORs (aORs) and 95% CIs. We performed all statistical analyses by using SAS version 9.3 (SAS Institute Inc., http://support.sas.com).

## Results

### Study Participants

All 959 participants were men (median age 35 years, age range 19–66 years). A total of 95.2% (913/959) (age range 29–43 years) were ethnic Han, and the remaining 4.8% (46/959) were from minority populations. A total of 46.8% (449/959) had a middle school education (9-year compulsory education practiced nationwide since 1986), 33.5% (321/959) had an education less than middle school, 13.8% (312/959) had a high school education (12 years), and 5.9% (57/959) had a college education or higher. A total of 45.2% (433/595) were local registered residents, and 54.8% (526/959) were migrants. BMI ranged from 14.9 to 33 (median 23.2, interquartile range 21.5–25.2).

A total of 14.1% (135/959) participants reported employment before current imprisonment, and the remaining 85.9% (824/959) were unemployed. A total of 16.3% (156/959) reported a contact history with TB patients and 16.8% (161/959) reported current conditions other than TB. A total of 28.8% (276/959) had a history of imprisonment before the current term, and the other 71.2% (683/959) were incarcerated for the first time. The current prison term ranged from 0.4 to 21.0 years (median term 3.9 years, interquartile range 2.6–5.4 years). A total of 59.5% (571/959) participants had >1 BCG vaccine scar.

### Comparison of Characteristics between Seropositive and Seronegative Participants

The rate of TB seropositivity was 52.0% (499/959; range 41.9%–60.9%) for all study participants for the 5 prisons with specific TB incidences (Table 1); the difference between seropositive and seronegative participants was significant (p<0.05). We compared frequencies of characteristics between seropositive and seronegative participants (Table 2). More LTBI-positive participants were >35 years of age; the difference between the 2 groups was significant (p<0.01). Positivity rates increased with age: <25 years, 18.9%; 25–34 years, 46.6%; 35–44 years, 58.5%; and >45 years, 68.6%. This increasing trend was significant (p<0.01).

**Table 1:** IGRA results for prisoners with specific TB incidences in prisons, Tianjin, China*

Prison no.	TB incidence†	No. (%) seropositive	No. (%) seronegative	Total	p value
1	1,945.2	106 (60.9)	68 (39.1)	174	<0.01
2	649.9	72 (41.9)	100 (58.1)	172	NA
3	892.7	91 (47.2)	102 (52.8)	193	NA
4	944.6	129 (57.6)	95 (42.4)	224	NA
5	603.0	101 (51.5)	95 (48.5)	196	NA
Overall	1,028.9	499 (52.0)	460 (48.0)	959	NA

*IGRA, interferon-γ release assay; NA, not applicable; TB, tuberculosis. †Reported pulmonary TB incidence (cases/100,000 persons) in the past 5 years was based on annual TB screening and case finding through daily healthcare seeking.

**Table 2:** Comparison of characteristics between IGRA-seropositive and IGRA-seronegative prisoners, Tianjin, China*

Characteristic	No. (%) seropositive	No. (%) seronegative	Total	p value by χ*^2^* test
Age, y				
<25	14 (2.8)	60 (13.0)	74	<0.01
25–34	197 (39.5)	226 (49.1)	423	
35–44	168 (33.7)	119 (25.9)	287	
>45	120 (24.0)	55 (12.0)	175	
Ethnicity				
Han	470 (94.2)	443 (96.3)	913	0.13
Other	29 (5.8)	17 (3.7)	46	
Education level				
Primary school	172 (34.5)	149 (32.4)	321	0.70
Middle school	233 (46.7)	216 (47)	449	
High school	94 (18.8)	95 (20.7)	189	
Residence				
Local	220 (44.1)	213 (46.3)	433	0.49
Migrant	279 (55.9)	247 (53.7)	526	
Employment				
No	425 (85.2)	399 (86.7)	824	0.49
Yes	74 (14.8)	61 (13.3)	135	
BMI†				
Abnormal: >25–<18.5	147 (29.5)	137 (29.8)	284	0.91
Normal: 18.5–25.0	352 (70.5)	323 (70.2)	675	
Contact history				
No	415 (83.2)	388 (84.3)	803	0.62
Yes	84 (16.8)	72 (15.7)	156	
Concurrent condition‡				
No	403 (80.8)	395 (85.9)	798	0.03
Yes	96 (19.2)	65 (14.1)	161	
*Mycobacterium bovis* BCG scar				
No	180 (36.1)	206 (44.8)	385	<0.01
Yes	319 (63.9)	254 (55.2)	573	
History of incarceration				
No	315 (63.1)	368 (80.0)	683	<0.01
Yes	184 (36.9)	92 (20.0)	276	
Current duration of incarceration, y				
<3.9	227 (45.5)	236 (51.3)	463	0.07
>3.9	272 (54.5)	224 (48.7)	496	
Facility-specific TB incidence, cases/100,000 persons§				
Low	173 (34.7)	195 (42.4)	368	0.01
Medium	220 (44.1)	197 (42.8)	417	
High	106 (21.2)	68 (14.8)	174	

*BMI, body mass index; TB, tuberculosis. †By World Health Organization standard (*13*).  ‡Chronic diseases, including diabetes, silicosis, hypertension, kidney disease. §The 2 low-incidence prisons reported 603.0 and 649.9 TB cases/100,000 persons, the 2 medium-incidence prisons 892.7 and 944.6 cases/100,000 persons, and the high-background prison 1,945.2 cases/100,000 persons.

The presence of factors other than TB, BCG scar, and history of previous incarceration was significantly higher for seropositive participants (p<0.05). As the level of TB incidence in the past 5 years for each prison increased, the rates of positivity also increased significantly (p<0.01): 47.0% positivity rate for the 2 prisons with low background incidence (603.0 and 649.9 cases/100,000 persons), 52.8% for the 2 prisons with medium background incidence (892.7 and 944.6 cases/100,000 persons), and 61.9% for the prison with high background incidence (1,945.2 cases/100,000 persons).

Longer duration (>3.9 years) in the current prison term was observed more often among positive participants than among negative participants; however, this difference was not significant (p = 0.07). Differences in ethnicity, education, residency, employment, BMI, and contact history were also not significant between the 2 groups (p>0.05).

### Factors Associated with LTBI

We identified several characteristics as factors associated with LTBI among prisoners (Table 3). The risk for LTBI increased with age, and from age <25 years to >45 years (aOR 1.7, 95% CI 1.4–2.0/10 years). A longer prison term led to a higher risk for LTBI (aOR 1.2, 95% CI 1.1–1.2 per year). A history of previous incarceration nearly doubled the risk for LTBI among prisoners. The risk for LTBI showed a major increase for prisons that had a higher incidence of TB in the past 5 years. Prisoners in prisons that had an annual TB incidence >1,900 cases/100,000 persons had a 1.9 times higher risk for TB than prisoners from 2 prisons who had an annual TB incidence of 600–650 cases/100,000 persons. Multivariate analysis showed a relationship between BCG scar and positive test results (aOR 1.3, 95% CI 1.0–1.8).

**Table 3:** Factors associated with LTBI seropositivity among prisoners, Tianjin, China*

Factor	OR (95% CI)	aOR (96% CI)	p value by type 3 test
All age groups	1.79 (1.53–20.9)	1.67 (1.42–1.96)	<0.01
Ethnicity			
Han	Referent	Referent	0.06
Other	1.61 (0.87–2.97)	1.90 (0.98–3.68)	
Education			
Primary school	Referent	Referent	0.76
Middle school	0.94 (0.7–1.25)	1.08 (0.79–1.48)	
High school	0.86 (0.6–1.23)	0.95 (0.63–1.44)	
Residency			
Local	Referent	Referent	0.15
Migrant	1.09 (0.85–1.41)	1.22 (0.93–1.62)	
Employment			
No	Referent	Referent	0.94
Yes	1.14 (0.79–1.64)	0.98 (0.65–1.49)	
BMI			
Normal: 18.5–25.0	Referent	Referent	0.52
Abnormal: >25–<18.5	0.98 (0.74–1.29)	0.91 (0.67–1.22)	
Contact history			
No	Referent	Referent	0.97
Yes	1.09 (0.77–1.54)	1.01 (0.70–1.46)	
Concurrent condition			
No	Referent	Referent	0.49
Yes	1.45 (1.03–2.04)	1.14 (0.78–1.67)	
*Mycobacterium bovis* BCG scar			
No	Referent	Referent	0.04
Yes	1.44 (1.11–1.86)	1.33 (1.01–1.75)	
History of incarceration			
No	Referent	Referent	<0.01
Yes	2.34 (1.75–3.13)	2.01 (1.48–2.74)	
Current duration of incarceration	1.11 (1.04–1.18)	1.15 (1.07–1.23)	<0.01
Facility-specific TB incidence, cases/100,000 population†			
Low	Referent	Referent	<0.01
Median	1.26 (0.95–1.67)	1.13 (0.83–1.53)	
High	1.76 (1.22–2.54)	1.87 (1.25–2.79)	

*BMI, body mass index; LTBI, latent tuberculosis infection; TB, tuberculosis. †The 2 low-incidence prisons reported 603.0 and 649.9 TB cases/100,000 persons, the 2 medium-incidence prisons 892.7 and 944.6 cases/100,000 persons, and the high-background prison 1,945.2 cases/100,000 persons.

## Discussion

Implementation of active case-finding and directly observed treatment using short-course chemotherapy in the prison system in Tianjin, China, led to a major decrease in pulmonary TB incidence, from 2,801.9 cases/100,000 persons (≈80 times the level in the general population) in 2000 to 735.1 cases/100,000 persons (≈30 times the level in the general population) in 2010 (*10*). This decrease confirmed the highly efficient strategy aimed at patients with active TB in crowded environments, which had a high incidence of TB. However, after this striking decrease, the level of TB incidence reached a plateau, still much higher in prisons than in the general population. Thus, prisons remain major TB reservoirs, indicating the limitation of strategies that focus merely on patients who have active TB. Early interventions before TB onset are required to achieve a further decrease in TB incidence in prisons, and management of LTBI in such crowded populations has been recommended by WHO (*11*).

Our study showed that the rate of LTBI among prisoners was >50%. This rate was much higher than that for the general population (13.5%–24.3%) in China, which was reported in recent studies that used IGRA (*14*–*16*). Two studies in Jiangsu Province reported IGRA positivity rates of 20.0% (1,060/5,305) and 24.3% (527/2,169) (*14*,*15*). Another study in Shenzhen reported an IGRA positivity rate of 17.9% (790/4,422) among rural migrant workers (*16*). A more representative multicenter study involving >20,000 participants in China reported an overall IGRA positivity rate of 18.8% (3,955/21,022) among all participants; the adjusted rates of LTBI by age and sex ranged from 13.5% to 19.8% in the 4 study sites (*17*). The higher rate of LTBI in prisoners than in the general population can be an attribution, as well as a consequence, of higher incidence of TB in prisons, which confirms the necessity of interventions for LTBI that target prisoners.

The rates of LTBI reported among prisoners varied in different studies, ranging from 11.7% to 92.5%, and were based mostly on results of the tuberculin skin test (TST). In Brazil, a study reported a TST positivity rate of 22.5% (620/2,752) for male prisoners and 11.7% (60/511) for female prisoners (*18*); these rates were lower than that for an earlier survey in a prison in the same country (49.3% [106/215]) (*19*). In other studies, rates of TST positivity were 17.9% (80/448) in Italy, 48% (204/425) in Pakistan, and 77.6% (643/829) in Colombia (*20*–*22*). In Malaysia, TST positivity rates were 84.7% (117/138) for HIV-infected and 92.5% (137/148) for HIV-uninfected prisoners in 1 prison; the overall positivity rate was 88.8% (254/286) (*23*). A study that used TST and IGRA in Taiwan reported a positivity rate of 24.6% (594/2416) for IGRA but >82% for TST among the same study participants (*24*).

Discrepancies reported for LTBI incidence in different studies might be related not only to testing methods, but also to the background of TB incidence and BCG vaccination among the general population. Although the rate of LTBI in our study was not directly comparable to those of most other surveys because of different testing methods used, this rate might have been higher if we had used TST instead of IGRA, which might have produced a rate similar to those reported in some other studies.

The risk for LTBI increases with age, regardless of whether TST or IGRA was used, among prisoners and in the general population. In the general population of China, the rate of LTBI detected by IGRA increased from 2.9% (43/1,459) for children 5–9 years of age to 32.4% (590/1,822) for persons >70 years of age (*17*). In 2 studies in China, the aOR of age to IGRA positivity was 1.1 (95% CI 1.1–1.1) per 10 years for migrant workers and 1.0 (95% CI 1.0–1.0) per 10 years for village populations (*14*,*16*). In our study, the aOR of age to LTBI was 1.7 (95% CI 1.4–2.0) per 10 years for prisoners, which demonstrated that the cumulative effect of age was even more pronounced for prisoners than for the general population.

The effect of age on TST positivity was also seen in prisons in other studies. In Italy, compared with persons <30 years of age, the aOR of age to TST positivity was 4.1 (95% CI 1.5–11.1) for persons 31–40 years of age and 3.8 (95% CI 1.4–10.6) for persons >40 years of age in prisons (*20*). In Barcelona, Spain, the aOR of age to TST positivity among immigrants entering prisons was 2.3 (95% CI 1.4–3.9) for prisoners >40 years of age compared with prisoners <40 years of age (*25*). In Pakistan, the aOR was 3.5 (95% CI 1.9–6.7) for prisoners >42 years of age compared with prisoners 18–26 years of age (*21*). The cumulative effect of age to LTBI suggests that being older can be a priority for implementing specific interventions against LTBI in prisons.

History of previous incarceration or duration of current incarceration was found to be a risk factor for LTBI in previous studies (*18*,*20*–*23*). In a prison in Malaysia, previous frequent incarceration was a risk factor for LTBI (aOR 1.2, 95% CI 1.0–1.4 for every previous incarceration) (*23*). In Italy, although previous imprisonment was not associated with TST positivity, current detention was an independent risk factor (aOR 1.1, 95% CI 1.0–1.2) (*20*). Previous incarceration or duration of current incarceration was also confirmed in studies in Pakistan, Colombia, Brazil, and Chile (*18*,*21*,*22*,*26*). Similar to previous studies, in our study, the history of incarceration and current duration in prison increased the risk for LTBI, which reflected cumulative TB transmission among prisoners. Therefore, LTBI screening and intervention should be prioritized in persons who had previous incarceration.

In this study, although the history of TB contact was not associated with LTBI, prisoners from the facility with the highest incidence of TB in the past 5 years had 1.9 (95% CI 1.3–2.8) times higher risk for LTBI. Similar findings were reported in a study from Colombia, in which contact history was not related to TST positivity (*22*). However, in that study, annual risk for LTBI varied between prison blocks with high and low incidences of TB; infection rates were 5.1% per year for blocks with a high incidence of TB and 2.7% for blocks with a low incidence of TB. Self-reporting of a contact history might be unreliable because of recall bias and variable personal understanding of TB. However, in the congested environment, prisoners could get LTBI without realizing it, and prisons with higher incidences of TB pose a greater threat of TB transmission. This finding suggests that early case finding and timely isolation of contagious TB patients should be intensified to reduce the risk for LTBI.

In this study, the prison with the highest incidence of TB once served as a quarantine facility in the city for contagious TB patients (sputum smear positive) found at entry screening (*10*). This facility also kept healthy inmates in separate wards, which might be the cause of the high incidences of TB and LTBI. After our survey, this situation was changed, and this prison now serves only as a quarantine facility and no longer receives healthy new inmates. This change was a positive step based partly on our TB surveillance and the LTBI survey.

Some studies have reported that LTBI was associated with being foreign-born (OR 4.9) and having a lower level of education (<5 years) (OR 1.90) (*20*,*21*). However, in our study, ethnicity, education, residency, employment before imprisonment, and BMI status were not associated with LTBI. Some studies that used IGRA reported a negative relationship between history of BCG vaccination and positive test results for TB, which was in contrast to results of studies that used TST (*19*,*24*,*27*). Two studies that used IGRA among general populations in China reported that a history of BCG vaccination was a protective factor against LTBI (aOR 0.8 [95% CI 0.7–1.0 in Jiangsu and 0.8 [95% CI 0.7–1.0 in Shenzhen) (*14*,*16*). However, 2 studies that used IGRA in a general population did not report an association between BCG scar and LTBI (*15*,*17*). In our study, BCG scar being a predictive factor of LTBI might be attributed to the age of the prisoners being older than the BCG protection period (75% of the prisoners were >29 years of age); alternatively, prisoners with a BCG scar might induce a better immune reaction in an IGRA, indicating a true TB infection. However, the effect of the aOR was marginal in this study, and the effect of BCG on LTBI among adults still requires stronger evidence.

Although prisons are recognized as major reservoirs of TB, control programs for TB in prison systems are facing several challenges (*7*). Our study was limited by safety concerns for prison administration. Thus, prisoners who committed severely violent crimes were not included in the study, and questions involving privacy and sensitive information, such as drug abuse, were not included in the questionnaire. In such situations, investigators had few opportunities to talk with study participants; instead, prison officers were in charge of quality control interpretation of information. Despite these limitations, the findings in this study provide useful information for control of TB in prisons in Tianjin and can be applied to other prisons.

In conclusion, LTBI prevalence is high in prisons in Tianjin, China. Previous incarceration and high facility–specific TB incidence are risk factors for LTBI. Age and duration of incarceration have cumulative effects on LTBI in prisons. High prevalence of LTBI increases the risk for TB incidence. Also, the high incidence of TB leads to high rates of LTBI. These findings suggest that preventive interventions to reduce LTBI before the onset of active TB among prisoners and to increase early detection of TB and timely quarantine of infectious case-patients will reduce transmission caused by overcrowding and poor ventilation.
